# Oncocytic papillary cystadenoma of the larynx: a case report

**DOI:** 10.1186/s13256-024-04425-2

**Published:** 2024-03-20

**Authors:** Alberto Caranti, Roberto Spasiano, Renato Piantanida, Salvatore Catalano, Ruggero Campisi, Manuela Bergmann, Matteo Trimarchi

**Affiliations:** 1 ENT & Audiology Department, University Hospital of Ferrara, Via Aldo Moro 8, 44124 Cona, FE Italy; 2 Gruppo Otorinolaringologico Della Romagna, Primus Medical Center of Forlì, Via Punta di Ferro 2/C, 47122 Forlì, FC Italy; 3 ENT Department, Ospedale Regionale Di Lugano, Ente Ospedaliero Cantonale EOC, Via Tesserete 46, 6900 Lugano, Tissin Switzerland; 4 USI - University of Italian Switzerland, Via Giuseppe Buffi 13, 6900 Lugano, Switzerland; 5 Institute of Pathology, Ente Ospedaliero Cantonale (EOC), Locarno, Switzerland

**Keywords:** Minor salivary glands, Minor salivary gland tumors, Ectopic minor salivary glands, Warthin’s tumor

## Abstract

**Background:**

Cystadenoma of the salivary glands is a rare benign clinical condition affecting both major and minor salivary glands equally. It constitutes approximately 2% of total neoplasms and 4.2–4.7% of benign formations in minor salivary glands. Typically presenting as a slow-growing, painless neoplasm, it can be distinguished from Cystadenolymphoma (Whartin’s Tumor) by the absence of lymphoid elements in histological examination. While mostly located in the oral cavity and oropharynx, it can also be found in sinonasal mucosa, and rare cases have been identified in the larynx.

**Case presentation:**

A 75-year-old Caucasian woman presented to the ear, nose, and throat department with complaints of dysphonia and headaches persisting for several months. Dysphonia had developed months after an unspecified vocal cord surgery elsewhere. Flexible laryngoscopy identified a left-sided cystic swelling affecting the supraglottic space, leading to respiratory obstruction and dysphonia. Head and neck computed tomography confirmed a 1.9 × 1.7 cm bilobed cystic mass originating from the left Morgagni ventricle. Microlaryngoscopy with CO_2_ laser excision and biopsy revealed a histopathological diagnosis of oncocytic papillary cystadenoma. Post-surgery, the patient fully recovered from dysphonia, with no significant complications noted. Long-term clinical surveillance was advised to detect potential recurrences promptly.

**Conclusion:**

Ectopic minor salivary gland tumors, both benign and malignant, should be taken into consideration as potential differential diagnosis for any swelling arising within the upper digestive tract mucosa. Ears, nose, and throat clinical examination completed by videolaryngoscopy can easily point out the location of the mass. Imaging is mandatory for differential diagnosis and for surgical planning. Surgical excision can provide both diagnosis and definitive cure.

## Introduction

The cystadenoma of the salivary glands represents a rare clinical entity that can affect both major and minor salivary glands with the same incidence [1]. It is a benign tumor of epithelial origin with a variable papillary component [2]. Often, a significant proportion of oncocytic cells is also present, leading to the inclusion of three variants in the current World Health Organization (WHO) classification of tumors: papillary cystadenoma, mucinous cystadenoma, and oncocytic cystadenoma [3]. Currently, the incidence of cystadenoma among minor salivary glands (MSGs) appears to be around 2% of total neoplasms and 4.2–4.7% of benign formations [4]. The most common site of incidence seems to be the lips, while it is rarely found on the palate [2]. It presents as a painless neoplasm, growing slowly, and therefore enters the differential diagnosis with cystoadenolymphoma [or Whartin’s Tumor (WT)], differentiating from it by the absence of lymphoid elements on histological examination [1]. The treatment of choice for this type of neoplasm is simple excision, complicated only by the possibility of recurrence in case of incomplete excision.

MSGs can be mostly found in the oral cavity and the oropharynx but can also be located in sinonasal mucosa, and a few have been detected in the larynx and trachea [5]. Furthermore, salivary tissue can be present in the head and neck region outside of the usual locations of major and minor salivary glands. This can be in the form of accessory salivary glands, in association with branchial cleft anomalies or, more rarely, as heterotopic salivary gland tissue, which is susceptible to the same disorders of the major and minor salivary glands, including infectious, inflammatory, and neoplastic diseases (both malignant and benign) [6].

In our article, we present a rare case of oncocytic papillary cystadenoma (OPC) of the larynx identified as a bilobed pseudocystic formation in a patient evaluated for worsening dysphonia. Laryngeal cystadenoma represents less than 1% of the total benign neoplasms found at this level, with approximately 150 cases reported in the literature [7].

## Case report

A 75-year-old Caucasian woman came to our attention at the ears, nose, and throat (ENT) department complaining of dysphonia and headache for several months. She had been discharged from the neurology department after a clinical investigation and magnetic resonance imaging (MRI) of the head were both negative. Anamnestic investigation revealed no risk factors, in particular no smoking, no alcohol assumption, and no environmental exposure. The patient revealed that her dysphonia arose some months after a non-specified previous surgical intervention on her vocal cords at another institution. No prior dysphagia or subjective dyspnea were reported.

At clinical examination, no significant alteration of the oral cavity was found, and no latero-cervical masses were apparent, but flexible laryngoscopy revealed a left-sided cystic swelling of the supraglottic space, with intact overhanging mucosa. The mass was around 2.0 cm in diameter and was laid on the glottic space, touching the left vocal cord, thus reducing the respiratory space and leading to dysphonia.

**Fig. 1:**
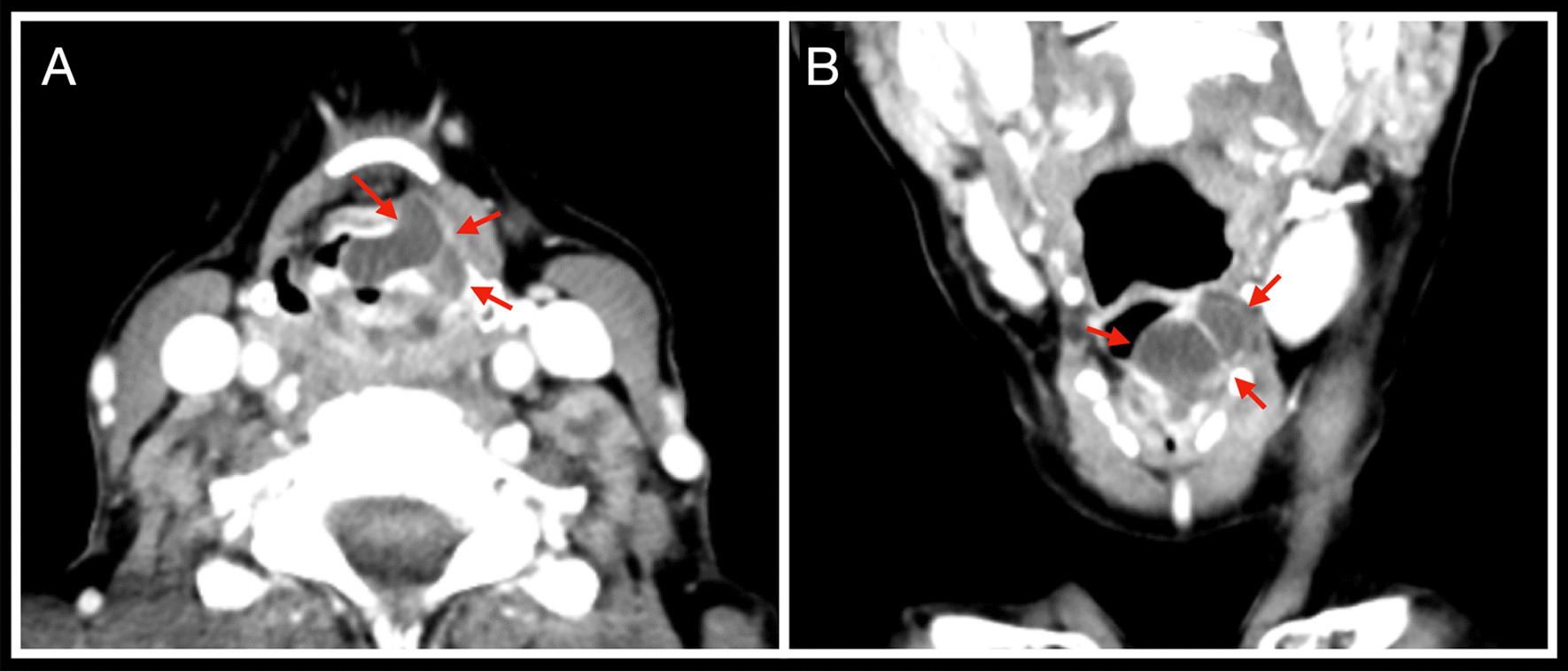
Head and neck CT showing a bilobed cystic neoformation rising from the left Morgagni ventricle. (**A**) Axial acquisition. (**B**) Coronal acquisition. The red arrows outline the borders of the lesion

**Fig. 2:**
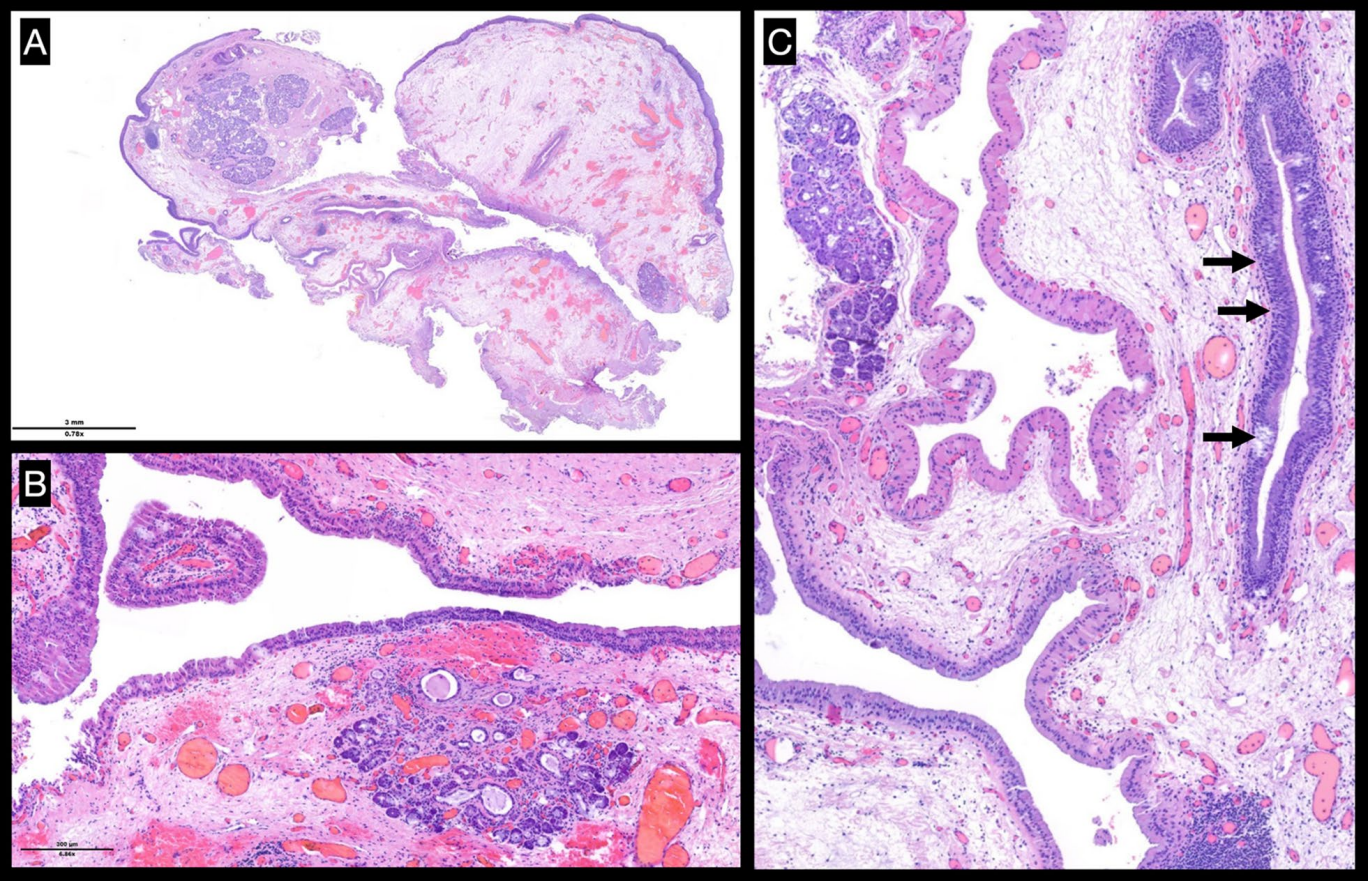
Histological examination: (**A**) Low-power view showing a multiloculated cystic lesion, well circumscribed but not encapsulated, with peripheral salivary gland tissue. (**B**) High-power view showing cystic lesion with peripheral entrapment of salivary gland tissue. (**C**) Focus on presence of respiratory epithelium (indicated by arrows)

**Fig. 3:**
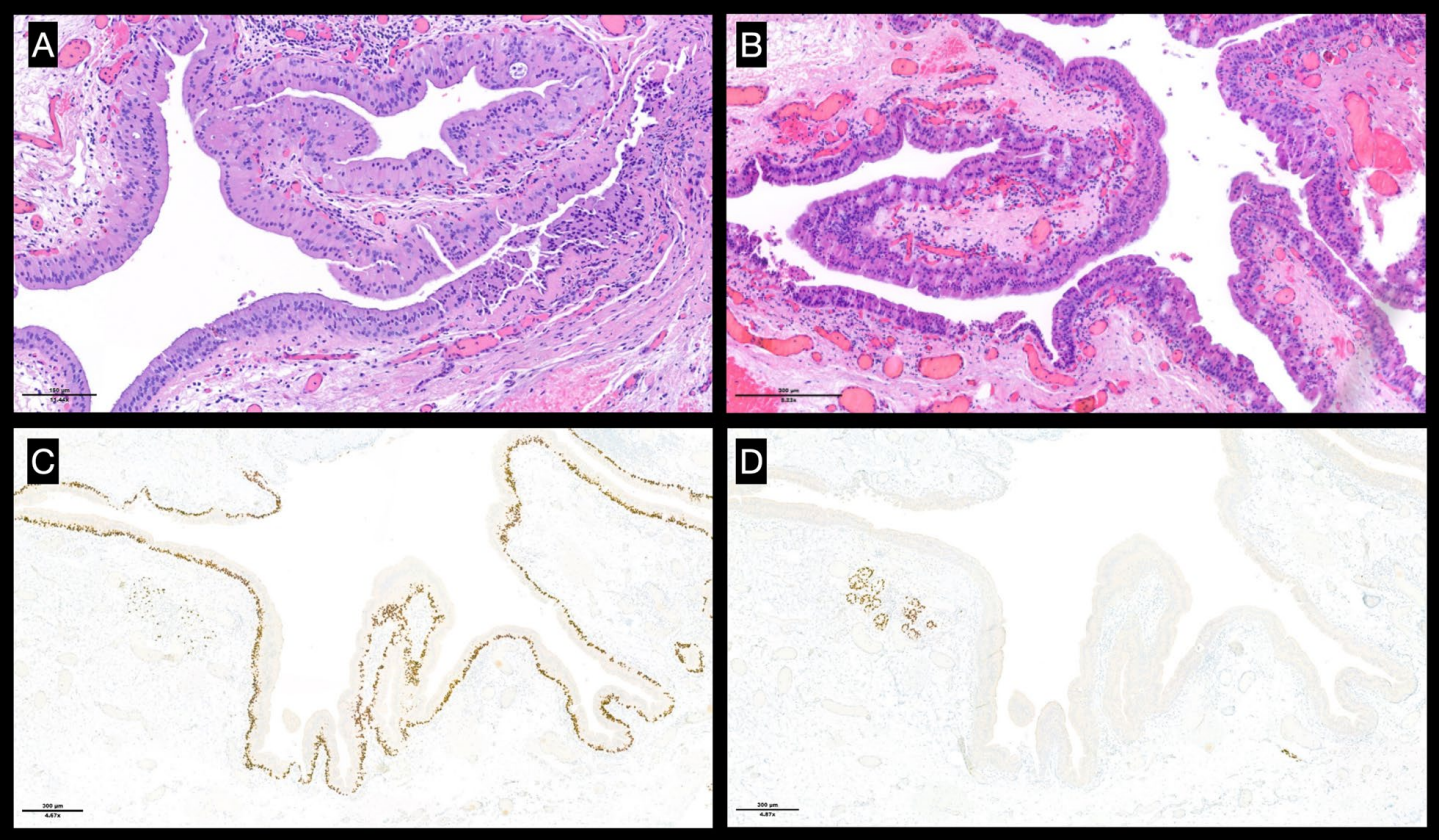
Histological examination: (**A**) high-power view showing simple papillary architecture lined by columnar epithelium in a double layer with oncocytic differentiation (similar to WT). Small collections of lymphocytes (patchy and limited) are present, but there is no lymphoid stroma (in contrast with WT). (**B**) There is no complex architecture, cytological atypia, mitotic activity, or invasive growth pattern. (**C**) p63 positivity highlights the basal cell layer at immunohistochemical stains. (**D**) Negativity for SOX10 and S100#

Computed tomography (CT) of the head and neck showed a 1.9 × 1.7 cm bilobed cystic neoformation rising from the left Morgagni ventricle and significantly reducing the respiratory space, which was laid on the glottic space and deepening into supraglottic parapharyngeal fat (Fig. 1). An under suspension microlaryngoscopy using CO_2_ laser for both excision and biopsy was performed. At histopathological examination (Figs. 2, 3), the mass was compatible with a MSG’s oncocytic papillary cystadenoma. No significant postsurgical complications were noted, and at 1 month after surgery the dysphonia completely recovered. The patient has been advised to remain under clinical surveillance for the following years to promptly recognize a potential recurrence.

## Discussion

MSGs range from 500 to 1000 small glands, with a diameter between 1 and 5 mm, located in the upper digestive tract [5]. Most are located in the oral cavity mucosa, especially in the lips, tongue, buccal mucosa, and palate. In contrast, ectopic localizations have been described in the literature, such as the oropharynx, tonsils, nasal cavity, paranasal sinuses, and even supraglottic space, larynx, and trachea [8]. As with the major salivary glands, the MSGs can be involved in benign and malignant tumors, but the absence of a specific International Classification of Diseases (ICD) classification and the large variety of histological types and localizations make their diagnosis and treatment challenging [8, 9]. It is well known that 80% of major salivary gland tumors are benign, while in MSGs the incidence of malignancy is similar, with a prevalence of malignant tumors close to 80% [10]. Regarding the epidemiology, the male-to-female ratio in minor and major salivary gland tumors is similar, with an overall ratio of 1.5:1 favoring women, reaching 1.6:1 for benign neoformations [11]. There is a similar prevalence of histologically benign tumors, where pleomorphic adenomas are the most common (65%), followed by WT (23%), recurrent pleomorphic adenoma (5.1%), and oncocytoma (2.8%). [12]. For ENT surgeons, knowledge about the potential location of MSGs and the main pathologies is fundamental in differential diagnosis. In fact, our patient presented at onset with an asymmetric laryngeal submucosal mass with no clear etiology. Submucosal laryngeal masses may present with hoarseness (frequent throat clearing), stridor, dysphonia, and dysphagia, or can be even non-symptomatic. These lesions can arise within the vocal folds (true and false) or pre-epiglottic and paraglottic spaces, or within the laryngeal cartilaginous framework. Diagnostic clinical evaluation is based on videolaryngoscopy, strobe-videolaryngoscopy, contrast-enhanced CT, and MRI, while definitive diagnosis is provided only by histological examination [13], keeping in mind potential ectopic MSGs and their pathologies are mandatory in differential diagnosis. Laryngeal masses may represent inflammatory or infectious disease, or primary benign or malignant tumors. MSGs can be involved in many different diseases such a primary tumor, a metastasis from a primary tumor in other major or MSGs, possible implantation of tumor after surgery, or a non-solid tumor localization (that is, lymphoma) [14]. After careful clinical examination, endoscopic exploration and imaging, as well as complete surgical excision with curative intent and obtaining a certain diagnosis, is advisable [14]. Ferlito *et al.*, considering the numerous reports of both benign and malignant neoplasms that may occur in heterotopic salivary tissue, reported that WT is the most common tumor affecting the ectopic MSGs [14]. The OPC is instead of much rarer occurrence, representing less than 1% of benign tumors of the minor salivary glands. Its occurrence in the larynx predominantly involves the supraglottic level (74% of cases), specifically affecting the ventricles rather than the false vocal cords [15]. Typically presenting as an isolated tumor, the treatment of choice is excision using CO_2_ laser. However, there are reported rare cases of very voluminous tumors or multiple localizations, which have necessitated open laryngectomy rather than total laryngectomy [15, 16].

## Conclusion

Ectopic MSG tumors, both benign and malignant, should be taken into consideration as potential differential diagnosis for any swelling arising within the upper digestive tract mucosa. For this purpose, ENT clinical examination completed by videolaryngoscopy (or even strobe-videolaryngoscopy) can easily point out the location of the mass. Imaging, such as CT and/or MRI, is mandatory for differential diagnosis and for surgical planning. In fact, complete surgical excision can provide both diagnosis and definitive cure. In our clinical case, after completion of surgery and histology, the mass was determined to be an OPC of a heterotopic salivary gland in the larynx, a rare tumor histotype that involves ectopic MSGs.

## Data Availability

All data generated or analyzed during this study are included in this article. Further inquiries can be directed to the corresponding author.
